# Furosemide-induced pseudoporphyria in a patient with chronic kidney disease: case report

**DOI:** 10.1590/2175-8239-JBN-2017-0029

**Published:** 2018-07-10

**Authors:** Giovana Memari Pavanelli, Sibele Sauzem Milano, Gabriela Sevignani, Juliana Elizabeth Jung, Vaneuza Araujo Moreira Funke, Marcelo Mazza do Nascimento

**Affiliations:** 1 Universidade Federal do Paraná, Complexo Hospital de Clínicas, Curitiba, PR, Brasil.; 2 Universidade Federal do Paraná, Complexo Hospital de Clínicas, Departamento de Nefrologia, Curitiba, PR, Brasil.; 3 Laboratório Diagnose, Curitiba, PR, Brasil.; 4 Universidade Federal do Paraná, Complexo Hospital de Clínicas, Departamento de Hematologia, Curitiba, PR, Brasil.

**Keywords:** Porphyrias, Renal Insufficiency, Chronic, Furosemide, Porfirias, Insuficiência Renal Crônica, Furosemida

## Abstract

**Introduction:**

Pseudoporphyria is a rare photodermatosis with characteristics similar to those of porphyria cutanea tarda, without, however, presenting abnormalities in porphyrin metabolism. Its etiology is related to chronic kidney disease, ultraviolet radiation and certain medications. The aim of the present study is to describe a case of furosemide-related pseudoporphyria in a patient with chronic kidney disease.

**Case description:**

A 76-year-old male patient with stage 4 chronic kidney disease and in continuous use of furosemide presented ulcerated lesions with peripheral erythema and central hematic crust in the legs. On a skin infection suspicion, treatment with quinolone and neomycin sulfate was initiated, without improvement. A biopsy of the lesion was performed, with histopathological examination demonstrating findings compatible with porphyria, although the patient did not present high porphyrin levels. The diagnosis of furosemide-induced pseudoporphyria was then established, with medication suspension, and there was a significant improvement of the lesions.

**Discussion:**

There are few cases of pseudoporphyria described, but it is believed that this condition is underdiagnosed, especially in patients with chronic kidney disease. Both clinical and histopathological findings closely resemble porphyria, differentiating it from normal levels of porphyrin in plasma, urine, or feces.

**Conclusions:**

Although the lesions are mostly benign, they may increase the morbidity and mortality of these patients, so a proper diagnosis and early treatment are extremely important.

## RESUMO

**Introdução:**

A pseudoporfiria é uma fotodermatose rara com características semelhantes às da porfiria cutânea tardia, sem, no entanto, apresentar anormalidades no metabolismo da porfirina. Sua etiologia está relacionada a doença renal crônica, radiação ultravioleta e determinados medicamentos. O objetivo do presente trabalho é descrever um caso de pseudoporfiria relacionada a furosemida em paciente portador de doença renal crônica.

**Descrição do caso:**

Paciente masculino, 76 anos, com doença renal crônica estágio 4 e em uso contínuo de furosemida, apresentou lesões ulceradas com eritema periférico e crosta hemática central nas pernas. Por suspeita de infecção de pele, foi iniciado tratamento com quinolona e sulfato de neomicina, sem melhora. Foi realizada então biópsia da lesão, com exame histopatológico demonstrando achados compatíveis com porfiria, sem, no entanto, o paciente apresentar níveis elevados de porfirinas. Foi então estabelecido o diagnóstico de pseudoporfiria induzida por furosemida, com suspensão de medicação , e houve melhora significativa das lesões.

**Discussão:**

Há poucos casos de pseudoporfiria descritos, mas acredita-se que essa condição seja subdiagnosticada, principalmente em pacientes com doença renal crônica. Tanto achados clínicos quanto histopatológicos se assemelham muito à porfiria, diferenciando desta por níveis normais de porfirina no plasma, na urina ou nas fezes.

**Conclusões:**

Embora as lesões sejam majoritariamente benignas, podem aumentar a morbimortalidade desses pacientes, por isso um diagnóstico adequado e tratamento precoce são de extrema importância.

## INTRODUCTION

Pseudoporphyria is a bullous photodermatosis with clinical and histological characteristics similar to those of porphyria cutanea tarda, but without abnormalities in porphyrin metabolism.^1^ It is an extremely rare disease in the general population, but may be a somewhat increased frequency in certain groups of risk.^2^ Among other causes, its etiology is associated with chronic kidney dialytic disease, exposure to ultraviolet radiation and to certain drugs. The drugs most frequently associated with pseudoporphyria are anti-inflammatory agents, especially naproxen, but also secondary to the use of antibiotics, antineoplastics and diuretics such as chlorthalidone, torasemide, bumetanide and furosemide.^3^ Except for photoprotection and suspension of possible medications involved, there is no specific treatment. This report aims to describe a case of furosemide-related pseudoporphyria in a patient with chronic stage 4-kidney disease.

## CASE PRESENTATION

A 76-year-old male patient previously diagnosed with systemic arterial hypertension and stage-4 chronic kidney disease with indefinite etiology, only reporting a previous history of kidney stones. He began follow-up at the oncology department for marginal zone splenic lymphoma. Initially, no specific treatment for lymphoma was instituted, and clinical follow-up was chosen.

**Figure 1:**
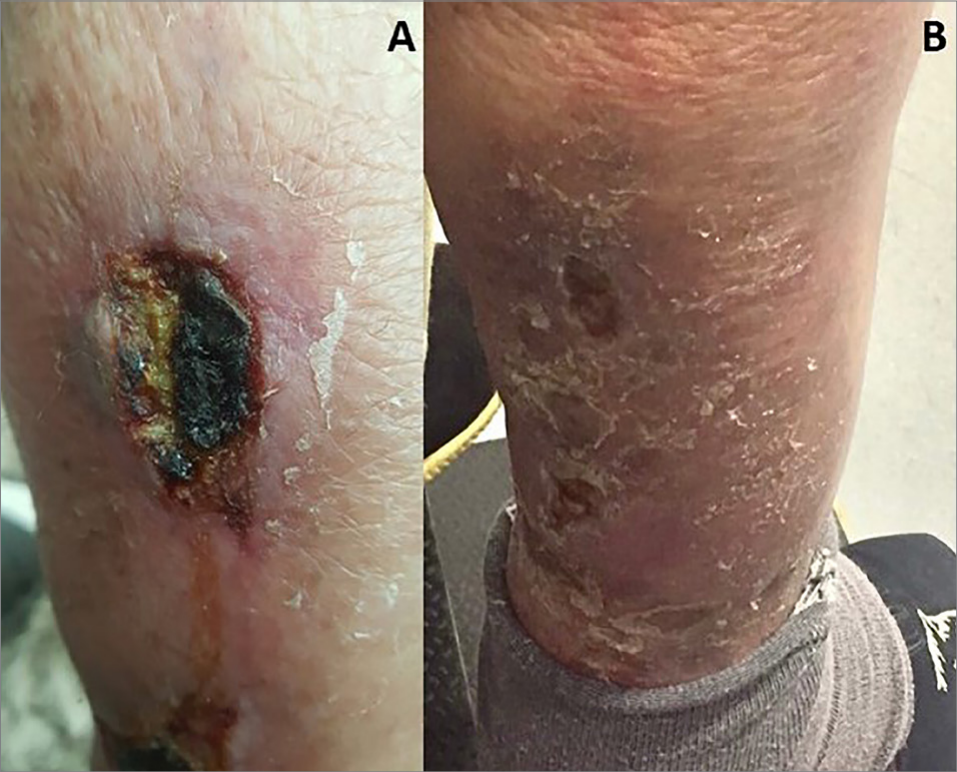
[A] Ulcerated lesion in the posterior region of the right leg, with hematic crust and pus, erythema and peripheral skin shedding, irregular borders; [B] Injuries in the healing process, with erythematous and scaly areas in the posterior region of the right leg.

Two months after diagnosis, two ulcerated lesions of approximately 10 centimeters in diameter appeared in the posterior region of the legs, with peripheral erythema and central hematic crust (Figure 1A). The patient reported local pain and heat, worsening at the end of the day and in orthostatic position. There were no lesions in other regions of the body, hyperpigmentation or hypertrichosis. He had been under furosemide for 3 months, potassium citrate and fluoxetine.

**Figure 2:**
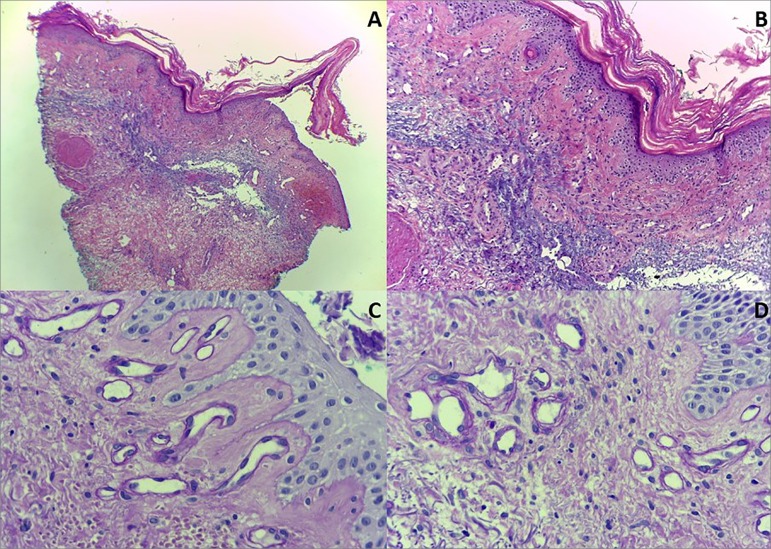
[A] HE 4X - Panoramic histological cross-section of the skin, with discrete lymphocytic infiltrate around the blood vessels; [B] HE 10 X - Histological skin cross-section with areas of epidermal atrophy and moderate solar elastosis in the dermis; [C] PAS 40 X - Histological cross-section of the skin in PAS staining with digestion demonstrating hyalinization of vascular walls on superficial dermis capillaries; [D] PAS 40 X - Histological skin cross-section on PAS staining with digestion demonstrating hyalinization of the vascular walls of the superficial dermis capillaries.

Due to the suspicion of skin infection, treatment with oral quinolone and topical neomycin sulfate was started, without improvements, and biopsy of the lesion was indicated. Histopathological examination revealed mild perivascular lymphocytic infiltrate and moderate solar elastosis, with hyalinization of vascular walls of capillaries on the superficial dermis with the periodic Schiff acid staining (Figure 2), findings it compatible with porphyria. Laboratory tests showed creatinine of 3.0 mg/dL, urea 144 mg/dL, calcium 11.5 mg/dL, Hb 9.0 g/dL, ferritin 1117 ng/mL and normal liver biochemistry. Urinary levels of porphyrin were normal - negative uroporphyrin at the 24-hour urine test - ruling out the diagnosis of porphyria.

Based on the histopathological findings associated with normal urinary porphyrin levels, the diagnosis of pseudoporphyria was established. The ailment was attributed to furosemide and the medication was discontinued. Five months after its suspension, there was a significant improvement in the lesions (Figure 1B).

## DISCUSSION

In 1964, Zelickson reported for the first time a case of pseudoporphyria related to nalidixic acid. After this first report, several other drugs have been associated with the disease, including diuretics.^1^^,^ 3 Pseudoporphyria has been reported in association with several diuretics, such as furosemide, torasemide, bumetanide, chlorthalidone and the combination of hydrochlorothiazide and triamterene. In addition, other factors, such as chronic kidney disease, dialysis and excessive exposure to ultraviolet radiation, were also associated with the onset of pseudoporphyria.^3^

There are less than 100 cases of pseudoporphyria described, but it is believed that the disease is not so uncommon, only underreported. In a study involving 363 hemodialysis patients, two presented pseudoporphyria. Few cases have been reported in patients with chronic non-dialytic kidney disease.^4^ The condition is probably underdiagnosed in patients with chronic kidney disease, who have a wide range of skin manifestations that are often poorly valued because they are multifactorial and may be related to electrolyte imbalance, buildup of uremic substances and comorbidities.^5^

The pathophysiological mechanism of pseudoporphyria is not well understood. It is believed that some drugs, such as furosemide, have a behavior similar to that of endogenous photoactivated porphyrins, acting on specific targets on the skin.^6^ Another proposed mechanism is that some products are deposited along the endothelium, leading to a response against the dermal microvascular endothelium. It is also believed that there are multiple mechanisms occurring, in addition to the immune response, the release of proteases that damage the endothelium.^7^ Therefore, some authors state that the most correct term for this condition is "therapy-induced photosensitivity", since it is a drug reaction with specific characteristics.^8^

The typical clinical presentation is the presence of blisters and vesicles on the hands, forearms, face, legs or feet. However, there are reports of cases that occurred without typical bullous presentation, with the development of crusts and ulcers, the diagnosis being confirmed by histological characteristics.^9^ Skin frailty and easy bruises after minor trauma are also common complaints.^10^ Pseudoporphyria, by contrast with porphyria, is rarely associated with hypertrichosis or hyperpigmentation.^3^

The histopathological characteristics resemble those found in porphyria. Examination of the lesions usually presents subepidermal vesicles with a discrete perivascular lymphocytic infiltrate - although this is not a necessary feature for diagnostic confirmation.^8^ The more specific findings of this condition include, in the periodic acid staining of Schiff (PAS), endothelial wall thickening and resistance to diastasis in the upper dermal microvasculature.^8^^,^ 11 These last two were evidenced in the anatomopathological examination of the patient's lesion, confirming the diagnosis of pseudoporphyria. In addition, in the biopsy in question, it was possible to observe histological findings compatible with solar elastosis, which is a marker of ultraviolet exposure, which corroborates the diagnosis.^12^

The diagnosis is based on normal levels of porphyrin in plasma, urine or feces in patients with clinical manifestations and histological findings similar to those found in porphyria. However, in the context of advanced chronic kidney disease, diagnosis may be difficult. In patients on hemodialysis or peritoneal dialysis, plasma porphyrin levels may be increased in the absence of enzyme deficiency, due to reduced excretion, making differential diagnosis between porphyria cutanea tarda and pseudoporphyria difficult.^13^ In addition, in non-dialytic patients, the definition of the disease's etiology can be particularly difficult, whereas there are few reported cases of pseudoporphyria in chronic pre-dialytic renal patients.

Treatment consists in the suspension of the possible medicines involved and in avoiding sun exposure. In cases associated with chronic kidney dialysis, prolonged use of N-acetylcysteine appears to be beneficial.^14^ Prognosis can be very good as long as the causative agent is identified and discontinued. However, the response is usually slow, with the possibility of complications by secondary infection. Improvements in the lesions is usually gradual, with complete resolution in a period of months to years.^15^

## CONCLUSION

Pseudoporphyria is a rare and probably underdiagnosed clinical condition; therefore, knowing its particularities is important for the differential diagnosis of skin lesions in patients with risk factors. Skin changes in patients with chronic kidney disease are poorly valued and, although often benign, may result in increased morbidity and mortality. Therefore, its recognition is essential for proper diagnosis and early treatment.
